# Blood pressure control and satisfaction of hypertensive patients following a switch to combined drugs of an angiotensin receptor blocker and a calcium channel blocker in clinical practice of nephrology

**DOI:** 10.1007/s10157-014-1017-7

**Published:** 2014-08-19

**Authors:** Hideki Kato, Takeshi Shiraishi, Shuko Ueda, Eiji Kubo, Tomoko Shima, Michito Nagura, Hirofumi Yano, Yuh Izumikawa, Masaru Shimada, Satoru Tomioka, Hitonari Nosaka, Kenichiro Kojima, Masayuki Tanemoto, Shunya Uchida

**Affiliations:** Department of Internal Medicine, Teikyo University School of Medicine, 2-11-1 Kaga, Itabashi-ku, Tokyo, Japan

**Keywords:** Angiotensin receptor blockers, Calcium channel blockers, Combination antihypertensive drugs, Hypertension, Blood pressure

## Abstract

**Background:**

Combination drugs containing an angiotensin receptor blocker and a calcium channel blocker have been widely commercialized in recent years, and their advantages, such as improvements in adherence, and reductions in medication costs, have been greatly emphasized. However, the actual situations and the impact of switching to combination drugs in clinical practice of nephrology are not fully understood.

**Methods:**

This study was conducted in outpatients of nephrology who received antihypertensive medicines, and who switched to combination drugs. Changes in the potency of the antihypertensive drugs, and blood pressure were examined retrospectively before and after changing treatments. In addition, the study also involved patients’ questionnaire, which examined changes in blood pressure at home, the presence or absence of missed doses, the impact on medication-related expenses, and the level of patients’ satisfaction with regard to combination drugs.

**Results:**

Survey results from 90 participants revealed that changing to combination drugs resulted in a reduction of missed doses, a decrease in blood pressure measured in an outpatient setting, and a reduction in medication-related expenses in total patients, non-chronic kidney disease (CKD) patients, and CKD patients.

**Conclusion:**

Our study shows that switching to combination antihypertensive drugs resulted in an improvement in adherence and a reduction in medication-related expenses, and revealed that patient satisfaction was high. Combination drugs for hypertensive patients may be beneficial in both medical and economical viewpoints.

## Introduction

Hypertension has the highest incidence among lifestyle-related diseases [1, 2] and is the most important among the major risk factors for cardiovascular and renal diseases [3]. The guidelines recommend that target blood pressure levels should be <140/90 mmHg, and <130/80 mmHg in patients with diabetes mellitus or renal disease [4]. Based on guidelines of hypertension in Japan (according to [5]), a blood pressure <140/90 mmHg is recommended for the elderly, and a blood pressure <130/80 mmHg is recommended in patients with diabetes mellitus, chronic kidney disease (CKD), or those recovering from a myocardial infarction [5].

Antihypertensive therapy extensively inhibits cardiovascular events [6], and the risks of developing stroke and ischemic heart disease decrease by 7 and 10 %, respectively, for each 2 mmHg decrease in systolic blood pressure (SBP) [7]; and the risks of stroke, ischemic heart disease, and overall mortality has also been reported to decrease by 14, 9, and 7 %, respectively, for each 5 mmHg decrease in SBP [8].

In recent years, various types of antihypertensive agents have been used in clinical practice; nonetheless, the number of hypertensive patients whose blood pressure levels <140/90 mmHg only accounts for 50 % in the United States, and 42 % in Japan [9, 10].

To achieve target blood pressure levels, various clinical guidelines recommend using angiotensin receptor blocker (ARB) as the first line because of its organ-protective effect, as well as calcium channel receptor blocker (CCB) because of its potency [4, 5].

Based on this background, combination antihypertensive drugs of ARB and CCB have been commercialized and widely used in clinical practice. However, much remains unknown about the situation of the patients whose drugs were switched to combination drugs. This study was conducted on outpatients with hypertension with or without CKD whose treatment was switched to combination drugs. We retrospectively examined the patients’ characteristics, clinical situations, physicians’ intention, and physicians’ judgments when conventional antihypertensive drugs were switched to combination drugs. Questionnaire survey was also conducted to reveal the patients’ satisfaction and missed doses.

## Methods

### Subjects

The study was conducted on hypertensive patients with or without CKD (non-hemodialysis patients), who visited the outpatient department of nephrology in Teikyo University Hospital. The study consisted of a retrospective survey in 90 patients whose antihypertensive medications had been switched to combination drugs containing ARB and CCB since December 2010. This study was conducted upon approval from the Ethics Committee of our hospital (Teikyo University Review Board, IRB #11-034) as well as oral and written consent from the patients. The study procedures were performed in accordance with the Helsinki Declaration.

### Switching treatment to combination drugs

At the time of this clinical trial, four different types of combination drugs containing ARB and CCB were on market in Japan. These drugs are Unisia LD (candesartan 8 mg + amlodipine 2.5 mg), Unisia HD (candesartan 8 mg + amlodipine 5 mg), Exforge (valsartan 80 mg + amlodipine 5 mg), Micamlo AP (telmisartan 40 mg + amlodipine 5 mg), Rezaltas LD (olmesartan 10 mg + azelnidipine 8 mg) and Rezaltas HD (olmesartan 20 mg + azelnidipine 16 mg). The decision of the switch and the selection of the combination drug were fully entrusted to the judgment of a physician in charge.

### Categorization of the potency of antihypertensive drugs

The antihypertensive potency of drugs was quantified based on the interview forms; a maximum dose of the standard doses was allocated as 1. The potency of the combination drug was calculated as a sum of the single antihypertensive drugs. Because the potency of diuretics is difficult to calculate, we excluded the patients whose treatments were switched to combination drugs containing diuretics or whose diuretic treatment had changed.

Table 1 shows the potency of the antihypertensive drugs that were used in the study.

**Table 1:** A list of antihypertensive drugs, drug potency and price

	Ingredients	Drug names	Dosage forms (mg)	Potency	Standard dosage (mg)	Prices (yen)
ARB	Candesartan cilexetil	Blopress	4	0.5	4–8	72.3
			8	1		140.4
			12	1.5		216.2
	Olmesartan medoxomil	Olmetec	10	0.5	10–20	68.2
			20	1		130.4
			40	2		197.9
	Valsartan	Diovan	40	0.5	40–80	61.4
			80	1		114.8
			160	2		223.7
	Telmisartan	Micardis	20	0.5	20–40	69.3
			40	1		131
			80	2		198.6
	Losartan potassium	Nu-lotan	25	0.5	25–50	75.5
			50	1		143.4
			100	2		217.3
	Irbesartan	Irbetan	50	0.5	50–100	68.5
			100	1		130.5
ACE inhibitor	Captopril	Captopril	12.5	0.33	37.5–75	21.5
	Alacepril	Cetapril	25	0.33	25–75	32.9
			50	0.67		58.8
β-Blocker	Bisoprolol fumarate	Maintate	2.5	0.5	5	70.6
			5	1		123
α-Blocker	Doxazosin mesilate	Cardenalin	1	0.25	1–4	32.9
			2	0.5		59.7
			4	1		113.3
CCB	Amlodipine besylate	Amlodin	2.5	0.5	2.5–5	31.1
			5	1		57.5
			10	2		87.5
	Benidipine hydrochloride	Coniel	2	0.5	2–4	31.3
			4	1		54.9
			8	2		113.3
	Cilnidipine	Atelec	5	0.5	5–10	33.9
			10	1		61.2
	Nifedipine	Adalat-CR	20	0.5	20–40	34.7
			40	1		65.1
	Azelnidipine	Calblock	8	0.5	8–16	36.9
			16	1		65.5
	Efonidipine hydrochloride ethanolate	Landel	10	0.25	20–40	21
			20	0.5		36.2
			40	1		67.7

	Ingredients	Drug name	Classes	Dosage forms of ARB and CCB (mg)	Potency of ARB and CCB	Price (yen)
Combination drugs of ARB + CCB	Candesartan cilexetil + amlodipine besylate	Unisia	LD	8 + 2.5	1.5	141.1
			HD	8 + 5	2	140.7
	Valsartan + amlodipine besylate	Exforge		80 + 5	2	1,203
	Telmisartan + amlodipine besylate	Micamlo	AP	40 + 5	2	133.2
	Olmesartan medoxomil + azelnidipine	Rezaltas	LD	10 + 8	1	84.7
			HD	20 + 16	2	158.1

### Blood pressure measurement

Each patient visited approximately at the same time (from 9 a.m. to 3 p.m.). Office blood pressure measurement was evaluated with an automated digital brachial artery blood pressure device (HEM-907, Omron, Japan) with patients in a sitting position. Blood pressures were measured three times and averaged for the evaluation before and at least 1 month after the switch.

### Questionnaire survey

A patient questionnaire survey was conducted after switch to the combination drugs. The questionnaire consisted of four items: increase or decrease in the frequency of missed doses, increase or decrease in the drug costs, changes in home blood pressure, and satisfaction of the combination drugs.

### Statistical analysis

Numerical data are presented as mean ± SD. Comparison between two groups was done by *t* test or paired *t* test as appropriate. Comparison among three groups was performed by ANOVA followed by Tukey HSD as post hoc analysis. For correlation analysis, Pearson’s or Spearman’s rho was utilized as appropriate. All statistical analyses were performed with IBM SPSS for Windows version 22 (IBM, Japan). *P* values <0.05 were considered as statistically significant.

## Results

### Patients

The antihypertensive medications of total 90 patients (58 men and 32 women; mean age 63.1 ± 13.4 years) were switched to combination of antihypertensive drugs containing ARB and CCB between December 2010 and February 2012. The baseline characteristics of the patients are shown in Table 2. SBP and diastolic blood pressure (DBP) were 142.7 ± 19.4 and 82.6 ± 13.0 mmHg, respectively, the values still above the target. The patients took 2.18 ± 0.59 types of antihypertensive drugs, and the mean potency was calculated as 2.22 ± 0.74. The components of the hypertensive drugs were ARB + CCB (*n* = 58, 64.4 %), ARB + CCB + diuretic agent (*n* = 11, 12.2 %), monotherapy using CCB (*n* = 9, 10.0 %), monotherapy using ARB (*n* = 4, 4.4 %), ARB + CCB + alpha-blocker + diuretic agent (*n* = 3, 3.3 %), ACE inhibitor + CCB (*n* = 2, 2.2 %), and others (*n* = 3, 3.3 %) (Table 2).

**Table 2:** Demographic data

Age (years)	63.1 ± 13.4
Sex	Male 58 (64.4 %) Female 32 (35.6 %)
CKD, No. (%)	42 (46.7 %)
SBP (mmHg)	142.7 ± 19.4 mmHg
DBP (mmHg)	82.6 ± 13.0 mmHg
Current antihypertensive medication, no. (%)	
ARB + CCB	58 (64.4 %)
ARB + CCB + diuretics	11 (12.2 %)
CCB	9 (10.0 %)
ARB	4 (4.4 %)
ARB + CCB + α-blocker + diuretics	3 (3.3 %)
ACEi + CCB	2 (2.2 %)
ARB + ACEi + CCB	1 (1.1 %)
ARB + CCB + α-blocker	1 (1.1 %)
CCB + diuretics	1 (1.1 %)
Months after the switch to combination drugs	
	4.2 ± 2.8 months

Forty-two patients (46.7 %) had CKD defined by the presence of proteinuria or an eGFR <60 mL/min/1.73 m^2^ calculated from an equation for the estimation of GFR in Japanese subjects [11].

### Changes in potency, number of tablets and drug costs

**Fig. 1:**
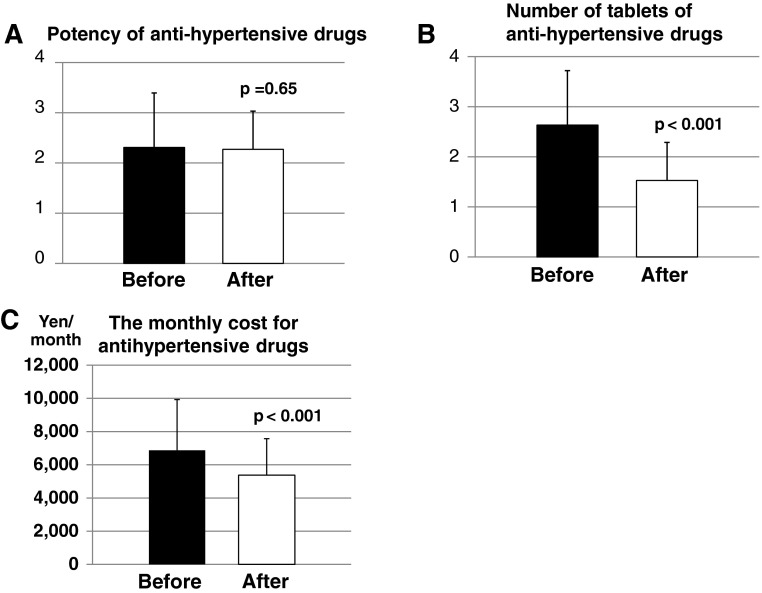
Changes in drug potency, number of tablets and drug cost by the switch to combination drugs. **a** Changes in drug potency. The potency did not change from 2.31 ± 1.09 to 2.27 ± 0.76 (*p* = 0.65). **b** Changes in the number of tablets of antihypertensive drugs. The number of tablets significantly changed from 2.63 ± 1.26 to 1.53 ± 0.91 (*p* < 0.001). **c** Changes in the monthly costs for antihypertensive drugs. The monthly costs significantly decreased from 6,873 ± 3,054 yen to 5,380 ± 2,198 yen (*p* < 0.001)

Changes in antihypertensive potency before and after the switch were examined. Fourteen patients (15.6 %) showed a decrease in potency after the switch; the group that showed no change in drug potency comprised 55 patients (61.1 %) and the group that showed an increase in drug potency comprised 21 patients (23.3 %). As a whole, the potency varied from 2.31 ± 1.09 to 2.27 ± 0.76 without a statistical significance (*p* = 0.65) (Fig. 1a). The average number of the tablets was changed from 2.63 ± 1.26 to 1.53 ± 0.91 (*p* < 0.001) (Fig. 1b). The changes in costs of antihypertensive drugs were estimated on the basis of the drug prices determined by the Ministry of Health, Labour and Welfare in Japan in 2012. The costs of antihypertensive drugs decreased in 68 patients (75.6 %) but increased in 21 patients (23.3 %). The average cost of antihypertensive medication per month changed significantly from 6,873 ± 3,054 yen to 5,380 ± 2,198 yen (*p* < 0.001), resulting in an average decrease of 18,167 yen per year (Fig. 1c).

### Changes in blood pressure

**Fig. 2:**
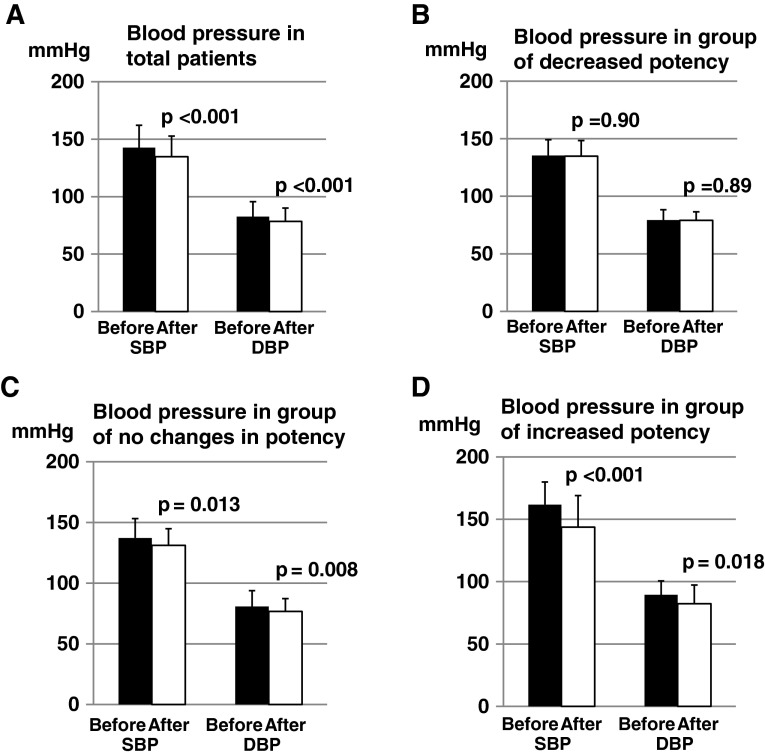
Changes in blood pressure after switching to combination drugs. **a** Changes in blood pressure in total patients. SBP (systolic blood pressure) significantly decreased from 142.7 ± 19.4 mmHg to 134.7 ± 18.0 mmHg (*p* < 0.001) and DBP (diastolic blood pressure) significantly decreased from 82.6 ± 13.0 to 78.4 ± 11.7 mmHg (*p* < 0.001). **b** Changes in blood pressure in the group of decrease in potency. SBP did not change from 135.4 ± 13.8 to 134.9 ± 13.5 mmHg (*p* = 0.90), and DBP did not change from 79.4 ± 8.9 to 79.1 ± 7.4 mmHg (*p* = 0.89). **c** Changes in blood pressure in the group of no change in potency. SBP significantly decreased from 137.2 ± 15.9 to 131.1 ± 13.7 mmHg (*p* = 0.013) and DBP significantly decreased from 80.8 ± 12.9 to 76.8 ± 10.6 mmHg (*p* = 0.008). **d** Changes in blood pressure in the group of increase in potency. SBP significantly decreased from 161.7 ± 18.2 to 143.6 ± 25.3 mmHg (*p* < 0.001) and DBP significantly decreased from 89.4 ± 11.2 to 82.3 ± 15.0 mmHg (*p* = 0.018)

In all 90 patients, the office blood pressure showed a significant decrease in both SBP (from 142.7 ± 19.4 mmHg to 134.7 ± 18.0 mmHg, *p* < 0.001) and DBP (from 82.6 ± 13.0 mmHg to 78.4 ± 11.7 mmHg, *p* < 0.001) (Fig. 2a). Next, we analyzed the changes in BP in association with the change in potency. In the group of decrease in potency (*n* = 14), neither SBP nor DBP significantly changed; SBP from 135.4 ± 13.8 to 134.9 ± 13.5 mmHg (*p* = 0.90), DBP from 79.4 ± 8.9 to 79.1 ± 7.4 mmHg (*p* = 0.89) (Fig. 2b). Even in the group of no change in potency (*n* = 55), SBP and DBP significantly decreased; SBP from 137.2 ± 15.9 to 131.1 ± 13.8 mmHg (*p* = 0.013) and DBP from 80.8 ± 12.9 to 76.7 ± 10.6 mmHg (*p* = 0.008) (Fig. 2c). In the group of increase in potency (*n* = 21), SBP significantly decreased from 161.7 ± 18.2 to 143.6 ± 25.3 mmHg (*p* < 0.001) and DBP significantly decreased from 89.4 ± 11.2 to 82.3 ± 15.0 mmHg (*p* = 0.018) (Fig. 2d).

We then examined the factors which correlated with the change in blood pressures. The changes of potency were significantly associated with the changes of SBP and DBP (Spearman’s *ρ* = −0.305, *p* = 0.003 and *ρ* = −0.247, *p* = 0.019). The decrease of the drug costs was also associated with the lowering of SBP and DBP (Pearson *r* = −0.291, *p* = 0.005 and *r* = −0.216, *p* = 0.041).

### Criteria for switching treatments to combined drugs

To examine how attending physicians switched the treatments, we compared the recipe before and after the switch. In most cases, combination drugs were chosen based on the ARB and CCB previously used. Patients who had already been using the same agents of ARB and CCB as those present in the combined drugs accounted for 36.7 % (*n* = 33). In this group, neither SBP (from 136.5 ± 20.1 to 135.1 ± 19.5 mmHg, *p* = 0.60) nor DBP (from 83.1 ± 13.9 to 80.2 ± 12.7 mmHg, *p* = 0.17) significantly changed. The potency did not change from 2.38 ± 0.80 to 2.31 ± 0.77 (*p* = 0.19) but the number of antihypertensive tablet dramatically decreased from 2.49 ± 0.78 to 1.33 ± 0.53 (*p* < 0.001) as well as the number of total tablets (from 5.51 ± 5.11 to 4.36 ± 4.80, *p* < 0.001), and costs of antihypertensive drugs appreciably decreased from 7,089 ± 2,114 to 5,697 ± 2,949 yen (*p* < 0.001). The second highest cases were the patients whose treatment had been switched or added on the basis of the ARB, and accounted for 28.9 % (*n* = 26). In this group, SBP decreased from 141.8 ± 19.0 to 133.4 ± 19.0 mmHg (*p* = 0.01) but DBP did not (from 79.7 ± 12.2 to 76.4 ± 11.1 mmHg, *p* = 0.15). The potency did not change from 2.73 ± 1.45 to 2.46 ± 0.88 (*p* = 0.20) but the number of antihypertensive tablet significantly decreased from 3.31 ± 1.79 to 2.08 ± 1.35 (*p* < 0.001) as well as the number of total tablets changed (from 10.1 ± 7.85 to 9.20 ± 8.28, *p* = 0.005), and costs of antihypertensive drugs also decreased from 8,569 ± 3,344 to 5,740 ± 1,869 yen (*p* < 0.001). The third highest cases were the patients whose treatment had been switched or added on the basis of the CCB; they accounted for 14.4 % of the cases (*n* = 13). In this group, SBP decreased from 152.0 ± 17.3 to 133.2 ± 17.9 mmHg (*p* = 0.02) as well as DBP (from 84.7 ± 14.0 to 75.7 ± 14.2 mmHg, *p* = 0.007). However, the potency did not change from 2.18 ± 0.97 to 2.19 ± 0.61 (*p* = 0.96). The number of antihypertensive tablet decreased from 2.46 ± 0.93 to 1.15 ± 0.36 (*p* < 0.001) but neither the number of total tablets (from 6.69 ± 3.93 to 5.77 ± 4.58, *p* = 0.053) nor the costs of antihypertensive drugs significantly decreased (from 5,698 ± 3,266 to 4,834 ± 1,252 yen, *p* = 0.33). In 20.0 % of the cases (*n* = 18), the treatment was switched to combined drugs which were unrelated to previous ARB or CCB. In this group, SBP decreased from 148.7 ± 13.4 to 136.2 ± 13.1 mmHg (*p* = 0.001) but DBP did not change (from 84.2 ± 10.8 to 79.9 ± 6.47 mmHg, *p* = 0.08). The potency increased from 1.67 ± 0.58 to 2.00 ± 0.53 (*p* = 0.018) and the number of antihypertensive tablet decreased from 2.10 ± 0.71 to 1.38 ± 0.59 (*p* < 0.001) as well as the number of total tablets (from 3.89 ± 2.81 to 2.94 ± 2.25, *p* < 0.001) but the costs of antihypertensive drugs did not change (from 4,876 ± 2,200 to 4,672 ± 971 yen, *p* = 0.68).

### Comparison of baseline characteristics between non-CKD and CKD patients

**Fig. 3:**
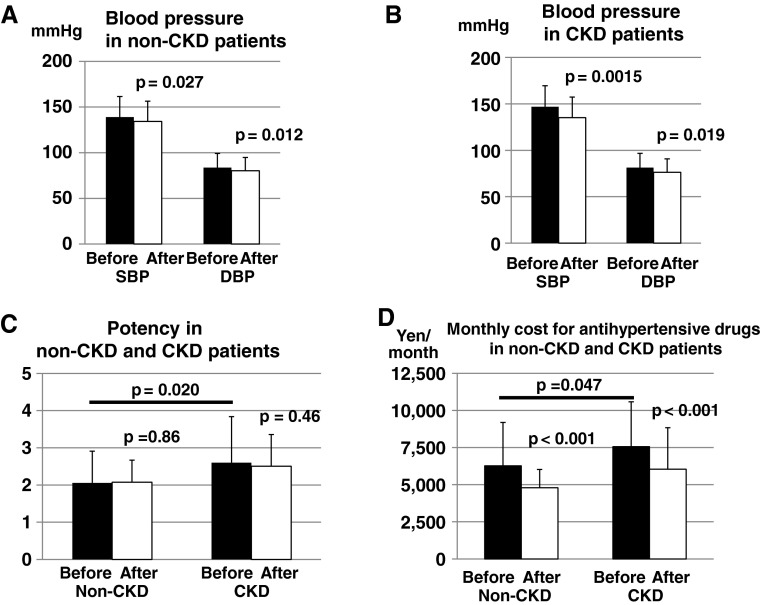
Comparison between non-CKD and CKD patients. **a**, **b** Changes in blood pressure in non-CKD and CKD patients. In non-CKD patients, SBP significantly decreased from 139.0 ± 15.1 to 134.3 ± 13.0 mmHg (*p* = 0.027) and DBP significantly decreased from 84.0 ± 10.3 to 80.3 ± 7.8 mmHg (*p* = 0.012). In CKD patients, SBP significantly decreased from 146.9 ± 22.5 to 135.2 ± 22.1 mmHg (*p* = 0.0015) and DBP significantly decreased from 81.3 ± 15.4 to 76.3 ± 14.5 mmHg (*p* = 0.019). **c** Changes in antihypertensive potency in non-CKD and CKD patients. The antihypertensive potency was higher in CKD patients than non-CKD patients (2.06 ± 0.85 in non-CKD vs. 2.60 ± 1.24 in CKD, *p* = 0.020). The potency did not differ significantly before and after the changes (from 2.06 ± 0.85 to 2.08 ± 0.60, *p* = 0.86 in non-CKD and from 2.60 ± 1.24 to 2.50 ± 0.85, *p* = 0.46 in CKD). **d** Monthly cost for antihypertensive drugs in non-CKD and CKD patients. The cost for the antihypertensive drugs was significantly higher in CKD patients than non-CKD patients (7,556 ± 3,024 yen in CKD vs. 6,276 ± 2,920 yen in non-CKD patients, *p* = 0.047) and were significantly decreased in both groups (*p* = 0.047)

We compared the baseline characteristics between non-CKD and CKD patients. CKD showed lower eGFR (75.3 ± 17.4 vs. 44.1 ± 22.8 mL/min/1.73 m^2^, *p* < 0.001), CKD patients showed slightly higher SBP (139.0 ± 15.1 vs. 146.9 ± 22.5 mmHg, *p* = 0.054) with the similar DBP (83.7 ± 10.3 vs. 81.3 ± 15.4 mmHg, *p* = 0.39) (Fig. 3a, b), even though antihypertensive drug potency was greater (2.06 ± 0.85 vs. 2.60 ± 1.24, *p* = 0.02) (Fig. 3c) and the number of antihypertensive tablets taken were higher in CKD patients (2.33 ± 0.92 vs. 2.98 ± 1.49 tablets, *p* = 0.015). The costs for the antihypertensive drugs were significantly higher in CKD patients than non-CKD patients (6,276 yen ± 2,920 yen in non-CKD patients vs. 7,556 yen ± 3,024 yen in CKD, *p* = 0.047) (Fig. 3d).

### Influence of the switch in non-CKD and CKD patients

In non-CKD patients, both SBP (from 139.0 ± 15.1 to 134.3 ± 13.0 mmHg) (*p* = 0.027) and DBP (from 84.0 ± 10.3 to 80.3 ± 7.8 mmHg) (*p* = 0.012) significantly decreased after the switch (Fig. 3a). In CKD patients, both SBP (from 146.9 ± 22.5 to 135.2 ± 22.1 mmHg) (*p* = 0.0015) and DBP significantly decreased after the switch (from 81.3 ± 15.4 to 76.3 ± 14.5 mmHg) (*p* = 0.019) (Fig. 3b).

In both non-CKD and CKD patients, the potency of antihypertensive drugs did not change significantly before and after the switch (from 2.06 ± 0.85 to 2.08 ± 0.60, *p* = 0.86 in non-CKD and from 2.60 ± 1.24 to 2.50 ± 0.85, *p* = 0.46 in CKD) (Fig. 3c). The number of antihypertensive tablets decreased significantly from 2.33 ± 0.92 to 1.32 ± 0.60, *p* < 0.001 in non-CKD but did not significantly decrease in CKD (from 2.97 ± 1.49 to 1.76 ± 1.13, *p* = 0.22). Urine protein in CKD patients tended to decrease but did not reach statistical significance (1.05 ± 1.21 to 0.92 ± 0.95 g/g creatinine, *p* = 0.06).

eGFR did not change either in non-CKD (75.3 ± 17.4 to 72.4 ± 15.9 mL/min/1.73 m^2^, *p* = 0.41) or in CKD patients (44.1 ± 22.8 to 39.4 ± 22.6 mL/min/1.73 m^2^, *p* = 0.73).

## Questionnaire survey

The following 4 items were asked in the survey.

A. Did missed doses decrease?
B. Did medication-related expenses decrease?
C. Did home blood pressure decrease?
D. Which do you prefer, the previous or the combination drug?

**Fig. 4:**
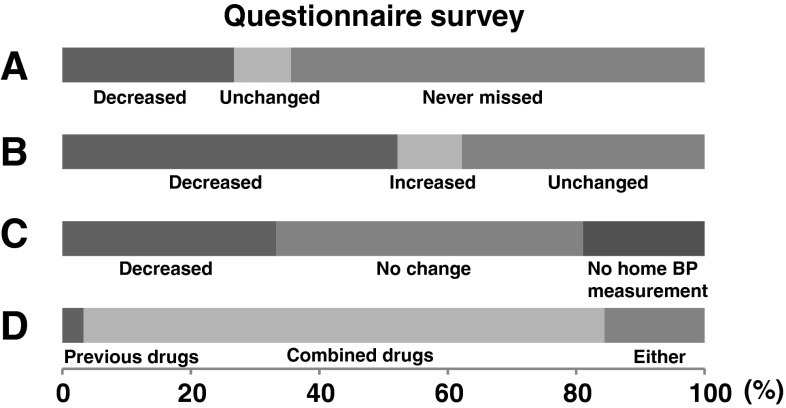
Questionnaire survey conducted after switching treatment to combined antihypertensive drugs.* A* Did missed doses decrease? 64.4 % (*n* = 58) answered, “I have never missed doses, even before switching treatment.” 26.7 % (*n* = 24) answered, “The number of missed doses has decreased.” 8.9 % (*n* = 8) answered, “The number of missed doses has remained unchanged.”* B* Did medication-related expenses decrease? 52.2 % (*n* = 47) answered that their drug costs had decreased; 37.8 % (*n* = 34) answered that their drug costs were unchanged; and 10 % (*n* = 9) answered that their drug costs had increased.* C* Did home blood pressure decrease? 33.3 % (*n* = 30) answered that their “home blood pressure decreased”; 47.8 % (*n* = 43) answered that there have been “no change”; and 18.9 % (*n* = 17) answered that they “did not measure their home blood pressure.”* D* Which do you prefer, the previous or the combination drug? 81.1 % (*n* = 73) answered that “the combined antihypertensive drugs are better”; 3.3 % (*n* = 3) answered that “the previous antihypertensive drugs are better.” and 15.6 % (*n* = 14) answered that “either is fine.”

All patients responded to the questionnaire and the result is shown in Fig. 4. In response to question A, 26.7 % patients (*n* = 24) replied that “missed doses have decreased” while 64.4 % (*n* = 58) answered that “never missed before” (Fig. 4A). In the group of decreased missed doses, SBP changed from 137.8 ± 16.5 to 132.5 ± 12.8 mmHg (*p* = 0.10), and DBP significantly decreased from 85.0 ± 12.3 to 80.0 ± 7.7 mmHg (*p* = 0.039). Even in the group that replied “never missed before,” SBP decreased from 142.6 ± 20.1 to 135.0 ± 20.1 mmHg (*p* = 0.004). However, the patients that replied “missed doses have decreased” did not necessarily showed the greater decrease in SBP or DBP (*p* = 0.69 by Spearman’s rho) probably because the patients who replied “missed doses unchanged” received relatively higher potency (0.25 ± 0.60 vs. −0.27 ± 0.98, *p* = 0.19 by Tukey HSD).

As for question B, 52.2 % of the patients (*n* = 47) replied that “medication-related expenses decreased” (Fig. 4B). Regarding question C, 33.3 % of the patients (*n* = 30) responded that “home blood pressure decreased”, whereas 47.8 % (*n* = 43) responded “no change” and 18.9 % (*n* = 17) responded that they “do not measure home blood pressure” (Fig. 4C). Regarding question D, 81.1 % of the patients (*n* = 73) answered that “they prefer the combination drug” and only 3.3 % (*n* = 3) answered that they “prefer previous drugs” (Fig. 4D).

## Discussion

Hypertension is the most frequently encountered disease in daily medical practice; however, the rate of achievement of target blood pressure levels is not always high [9, 10]. The use of combination drugs has been advocated due to an improvement in adherence, leading to the achievement of target blood pressure and decrease in the incidence of cardiovascular events [12, 13]. However, there have been virtually no clinical reports how antihypertensive drugs are replaced with combination drugs and what outcomes are obtained after the switch. Our present results revealed several findings.

The first finding is that the largest number of patients was the category of “no change in drug potency” after switch to combined formulation. This suggests that in most cases, the contents of the antihypertensive drugs themselves are left unchanged. The group with the second largest number of patients was the category of “increase in drug potency”. Interestingly, this group had higher blood pressure before switching treatment, revealing that switch was also intended to increase in potency in these cases.

Secondly, in our study, most of the patients took less than three kinds of oral antihypertensive drugs. According to the ALLHAT study, approximately 30 % of patients with blood pressure controlled at 140/90 mmHg or lower were reported to be taking at least 3 different types of drugs orally [14]. According to the CRIC study, 32 % of CKD patients were reported to be taking at least 4 different types of drugs orally [15]. Our findings showed that while patients taking more than 4 different oral antihypertensive drugs are frequently seen in daily clinical practice, these patients are not selected to switch to combined drugs.

We also examined how the combination drugs were selected and used by each physician. The findings showed that in many cases, the patients had already been using the same ARB and CCB included in the combined drugs or the combined drugs included the same ARB which patients had already used. This may reflect the fact that antihypertensive therapy had been conducted with a focus on ARB, as recommended by various guidelines pertaining to hypertension.

In this study, a significant decrease in blood pressure was found not only in the group that showed an increase in potency but also in the group in which potency remained unchanged. This decrease was probably due to an improvement in adherence. Adherence to medication is known to have an impact on blood pressure control, and patients often hesitate to take their oral medication when the number of tablets is large [16]. Our results suggest that the reduction in the number of drugs and beginning a treatment using new drugs might have caused improvements in both adherence and blood pressure.

From the perspective of medical economics, our survey also suggested that switching to combination drugs may lead to a reduction of medical expenses. Based on previous reports, combination therapy using both ARB and CCB has also been shown to be more cost-effective in treating hypertension than monotherapy using CCB or ARB [17]. The prices of combined drugs containing ARB and CCB have been set as low as approximately 70 % of the total price of each monotherapy, thus switch to combination drugs could be even more cost-effective. However, since our study included the patients whose medical costs are totally covered by government, thus this might explain the discrepancies between the ratio of patients with decreased cost and ratio of patients who answered “medication-related expenses decreased”.

In our patients, no major adverse effects were observed, including severe hypotension, rapid deterioration of renal functions, and electrolyte disorders. That might be due to the fact that most of the patients’ antihypertensive potency did not change between before and after the switch. In this regard, mixed formulations containing ARB and CCB might be safe when switching treatment.

There are several limitations in the present study that need to be taken into consideration. First, the study was not a parallel comparative study between a group that had switched treatment to combination drugs and a group that had not. Thus, the evidence level is not high enough, but our study vividly revealed the actual situations of clinical practice especially in nephrology. Next, switching to combination drugs was entrusted to the attending physician’s judgment and choice, which might create some bias. However, by surveying retrospectively, we could successfully reveal the physician’s attitude in clinical practice. The third limitation was related to the questionnaire survey. Blood pressure, adherences and antihypertensive potency were expressed as numerical values, whereas the level of satisfaction was subjective. There is a method using an analog scale, but in the present study, there was no need to do so. Final limitation was the method used in the calculation of the antihypertensive potency. The issue is whether a comparison of the antihypertensive effects belonging to different classes is possible or not. However, when antihypertensive drugs are released in the market, the doses are determined on the basis of their antihypertensive effects, thus our methods of quantification might not be precise but sufficient for comparison.

Despite these limitations of our study, the present results clearly show that switch to combination drugs is of high clinical utility from the perspective of blood pressure control, adherence, health economics, and patient’s satisfaction.
